# Long standing biliary colic masking chylous ascites in laparoscopic roux-en-Y gastric bypass; a case report

**DOI:** 10.1186/s12893-018-0374-7

**Published:** 2018-06-19

**Authors:** Louai R. Zaidan, Elhaitham K. Ahmed, Bachar Halimeh, Yasser Radwan, Khalil Terro

**Affiliations:** 1 Alfaisal University, Riyadh, Saudi Arabia; 2 Specialized Medical Center Hospital, Riyadh, Saudi Arabia

**Keywords:** LRYGB, Chylous ascites, Internal hernia, Biliary colic, Abdominal pain

## Abstract

**Background:**

Chylous ascites is considered to be an intra-abdominal collection of creamy colored fluid with triglyceride content of > 110 mg/dL. Chylous ascites is an uncommon but serious complication of numerous surgical interventions. However, it is a rare complication of LRYGB. An internal hernia limb defect is thought to be the underlying etiology, where the hernia will cause lymphatic vessel engorgement and lymphatic extravasation.

**Case presentation:**

We report a case of a 29 years old male with a 9 year history of laparoscopic Roux en y gastric bypass (LRGYB), presenting with recurrent abdominal pain for 2 months radiating to the right shoulder. Ultrasound examination revealed gallstones and the patient was subsequently admitted for laparoscopic cholecystectomy. Intraoperatively, whitish colored fluid, high in triglycerides content was aspirated. During exploration, an internal hernia limb defect was found and corrected.

**Conclusion:**

Post LRGYB patients with symptoms of recurrent abdominal pain should be suspected for chylous ascites reflecting an internal hernia.

## Background

Chyloperitoneum is defined as the accumulation of lymphatic fluid in the peritoneum due to leakage/break in the lymphatic system. Three reasons have been thought to cause this accumulation: (1) Obstruction of the vessels by a mass or tumor, leading to exudation through dilated lymphatic vessel’s wall or rupture of the vessel. Usually, these patients develop protein losing enteropathy. (2) Formation of a fistula between the lymphatic vessels and the peritoneum or mesentry secondary to adhesions, trauma or surgery. (3) Leakage from the retroperitoneal megalymphatics with or without an evidence of fistula, usually as a consequent of congenital diseases. In order to define ascetic fluid as chyle, the triglyceride content of the fluid must be greater than 110 mg/dl (2 to 8 times higher than plasma levels) and its specific gravity greater than 1.012. It commonly has the appearance of a turbid, milky fluid with no odor [1].

Chylous ascites has been reported to be most commonly due to malignancies in adults. In post-surgical cases, it has been most related to either vascular or lymphatic related operations [1]. However, only five cases have been reported in post-bariatric surgeries, three of which were after Roux en Y gastric bypass, all of them presenting 12–24 months later in in the mid post-op period. [2–5] We are reporting a fourth case of chylous ascites, interestingly presenting 9 years after LRYGB.

## Case presentation

A 29-year-old male, with a known surgical history of LRYGB 9 years ago, presented to the emergency department complaining of sharp, severe abdominal pain in the right upper quadrant (RUQ). The pain began two months ago in an intermittent fashion that would occasionally radiate to his right shoulder and progressively became worse with time. The patient did not report any constipation, distension, or vomiting despite feeling nauseated during these attacks. The pain was not related to any movement or meals and it was moderately relieved by over the counter analgesia.

Ten years ago, the patient was diagnosed with obstructive sleep apnea (OSA), through a sleep study, due to morbid obesity. He then was advised to undergo LRYGB as a treatment. He does have any other medical problems. Family history is notable for diabetes mellitus and hypertension but no history of hyperlipidemia or obesity. His body mass index (BMI) prior to the surgery was 49.3 Kg/m^2^. He underwent a successful LRYGB with no complications or readmissions. After the surgery for a year and a half, he followed up with his primary surgeon. During that time, he lost 75 Kg, reaching a BMI of 26.9 Kg/m^2^. He then was able to maintain his weight following the operation, but failed to continue to follow up for the past 2 years. In those 2 years, he gained weight, raising his BMI to 29.9 Kg/m^2^, after which he modified his diet to reduce his BMI to 23.9 Kg/m^2^. This weight reduction was achieved prior to his presentation.

On physical examination, the patient was afebrile with normal vital signs. Examination of his abdomen revealed moderate tenderness over the epigastrium, central abdomen, and right hypochondrium with a positive Murphy’s sign. His laboratory investigations, including complete blood count, urine analysis, liver and renal function tests, were all within normal limits. An ultrasound examination revealed two gallstones and upper borderline diameter of common bile duct, raising the suspicion of biliary obstruction.

Correlating his investigation results with his symptoms, he was admitted and scheduled for a cholecystectomy. Formal laparoscopic cholecystectomy was carried out the next morning. During exploration, twisting of the common channel was observed, indicating an internal hernia, although the patient’s complaint did not suggest internal hernia in the differential diagnosis. The internal hernia was identified but reduction was not feasible through laparoscopy. Mini midline laparotomy was subsequently done for reduction of the hernia and more detailed exploration. The hernia was through the mesenteric defect between the alimentary limb and the biliopancreatic limbs. Both limbs of the anastomosis were identified and complete reduction of the internal hernia was done, in addition to suturing of the defect found in the mesentery. No other defects were found. The small intestine’s mesentery showed prominent whitish colored vessels. Whitish thick fluid was noticed, accumulating in the right paracolic gutter and in Morison’s pouch. All of the fluid was then aspirated, measuring a total of 752 mL. Analysis of the fluid showed a triglyceride level of 534 mg/dL and a Lactate Dehydrogenase (LDH) level of 512 U/L. A classic Blake drain 24 F was inserted into the pelvis. The patient passed an uneventful post-operative period. On Postoperative Day (POD) one, fluid collected from the drain was 440 ml, chylous in appearance, while on POD 2–4 the drainage was serous in appearance. The drain was removed on POD 4. The patient was discharged on POD 4 after being able to ambulate independently on his own and eat and drink with no complications.

The patient did not return back for his follow-up visits and when he was called by the team, he confirmed that he has no complains up to this day.

## Discussion and conclusion

Obesity is still considered a major burden worldwide. Obesity is defined as a BMI > 30 kg/m2. The World Health Organization (WHO) has reported that in 2016, 13% of the world’s adult population were obese, with obesity prevalence doubling between 1975 and 2016 [6]. Obesity is an extensively spread modifiable risk factor for several diseases including diabetes mellitus, cardiovascular disease, joints disease, etc. [7, 8].

Several approaches have been addressed worldwide to treat obesity and prevent its complications. However, lifestyle modifications and medical therapy are not always effective, especially in cases involving morbid obesity. Therefore, bariatric surgery is an indication for treating obesity with a BMI above 40 kg/m2 or a BMI above 35 kg/m2 with comorbidities, according to the latest consensus guideline by the American Society for Metabolic and Bariatric Surgery (ASMBS). Of the various bariatric surgeries, LRYGB is considered the gold standard in treating obesity. Nevertheless, like any surgical procedure, LRGYB has a risk for developing several complications, several of which require repetitive follow-up postoperatively [7–9].

Internal hernia is one of the most common complications of LRYGB [10]. The prevalence of internal hernia has reached up to 14% [11]. Small bowel obstruction is the most feared complication, as it is considered an emergency and is most likely due to an internal hernia [12]. A review by Koppman et al. reported an incidence of small bowel obstruction in 0.6–11% [13]. Ghandi et al. reported that 3.8% of LRYGB patients suffered from small bowel obstruction, with 55% of obstruction cases being a result of internal hernia [14]. Internal hernia is frequently a result of the loss of the mesenteric fat, which provides protection and occupation of space to limit the movement of the small bowel, presenting in a delayed fashion. Moreover, fewer adhesions are formed from laparoscopic surgeries compared to open procedures, allowing more space for small bowel movement [15].

It was reported from a review of several studies that the incidence of internal hernia after a LRYGB was between 0.2–8.6%. The most common presentation was small bowel obstruction symptoms, ranging from 1.8–9.7% after a LRYGB [16].

Typically, patients with internal hernia have a chronic recurrent presentation of colicky abdominal pain, nausea and vomiting. The presence of an internal hernia increases the risk of developing small bowel obstruction as a sequela, usually presenting in a late stage [9].

In our case, the patient presented with the typical symptoms of biliary colic, which was supported by the presence of gallstones on ultrasound. This presentation masked the real patient’s problem; an internal hernia leading to chylous ascites.

Internal hernia can occur due to a surgical defect, depending on whether a retrocolic or antecolic approach is utilised. Petersen’s defect is the presence of space between the Roux limb and the transverse mesocolon. A second defect might also be present between the biliopancreatic and Roux limbs at the jejunojejunostomy. In cases of retrocolic approach, a third defect in the transverse mesocolon might be created. This is the most common reported site of internal hernia, prompting several surgeons to adopt the antecolic technique to eliminate such defect. In addition, the latter is consistent with an intermittent internal hernia that has the potential to reduce itself spontaneously, which explains the recurrent pain that our patient was having. Review of the literature supports the antecolic technique, as lower rates of herniation were reported when compared to the retrocolic technique. Most of internal hernias can be successfully treated laparoscopically, through the reduction of the herniated limb and the closure of the mesenteric defect. However, due to the fact that laparoscopy procedures possess a lower risk of adhesion formation, this can contribute to the risk of hernias by allowing more intestinal movement around a potential mesenteric space. Thus, laparotomy may help reduce the risk of developing internal hernia, as a result of more adhesions presence. [17].

Up to our knowledge, only three cases of chylous ascites post LRYGB were reported. An average time for presentation with chylous ascites in these cases was 20 months [2, 4, 5]. In our patient, the presentation was after 9 years.

Hidalgo et al. reported a 40-year-old morbid obese patient with comorbidities of exertional dyspnea and joint disease who underwent a LRYGB 15 months prior to his presentation. He presented with mild chronic abdominal pain and nausea. A computed tomography showed intra-abdominal fluid collection. The patient was taken for a diagnostic laparoscopy where a common channel internal hernia was identified and corrected. In addition, milky fluid was extracted and analyzed, with reports showing high triglycerides levels [2].

Hanson et al. reported a 22-year-old woman presenting with severe abdominal pain 2 years after undergoing LRYGB, during which she lost 90lbs. Computed tomography showed a huge collection of mesenteric free fluid, necessitating an urgent operation. Intraoperatively, a mesenteric defect was identified, in which the common channel was fully herniating through. 4 l of free fluid was extracted with the analysis reporting a triglycerides level of 388 mg/dL [4].

Capristo et al. reported a 61-year-old heavy smoker and heavy alcohol drinking man who underwent a LRYGB 18 months prior to the presentation of a sudden weight gain of 5 Kg, abdominal distention and shortness of breath. His BMI was reduced from 41.8 kg/m2 to 31.7 kg/m2. On physical examination, the patient had significant ascites, right pleural effusion and left supraclavicular lymph node enlargement. Paracentesis and thoracentesis evacuated a total of 4.5 L of whitish fluid, high in triglycerides. However, lymph node biopsy, computed tomography and positron-emission tomography revealed a poorly differentiated gastric carcinoma. This finding indicated that the chylous ascites was not related to the LRYGB [5].

These cases along with our case showed that chylous ascites is a manifestation of an internal herniation from a mesenteric defect after a LRYGB. These cases might present with vague or misleading symptoms.

Physicians, including general practitioners and emergency doctors, should have a high index of suspicion for internal hernia for post LRYGB patients, even if they present with mild to moderate symptoms of abdominal colicky pain. A delayed diagnosis of internal hernia increases the risk of developing acute small bowel obstruction. Such cases should be transferred to bariatric surgery if possible or undergo laparoscopic exploration, as it is the most sensitive and specific diagnostic tool, compared to imaging such as Computed Tomography (CT) scan which does not help in diagnosis [18].

In conclusion, our case was about a patient who developed chylous ascites 9 years after he underwent a LRYGB. While the most common cause of chylous ascites in adults is malignancy, it can also be an unusual presentation due to internal herniation that arises from a mesenteric defect post LRYGB. Chylous ascites in these case present with very mild and vague symptoms that might deviate the differential diagnosis away from a possible internal hernia. Thus, chylous ascites should be suspected in all LRYGB patients presenting with mild abdominal symptoms and properly managed accordingly.

## Abbreviations

ASMBS: American Society for Metabolic and Bariatric Surgery
BMI: Body Mass Index (Kg/m2)
CT: Computed Tomography
LDH: Lactate DeHydrogenase
LRYGB: Laparoscopic Roux-en-Y Gastric Bypass
OSA: Obstructive Sleep Apnea
POD: Post-Operative Day
RUQ: Right Upper Quadrant
WHO: World Health Organization
